# Evolution of rumen and oral microbiota in calves is influenced by age and time of weaning

**DOI:** 10.1186/s42523-021-00095-3

**Published:** 2021-04-21

**Authors:** Nida Amin, Sarah Schwarzkopf, Asako Kinoshita, Johanna Tröscher-Mußotter, Sven Dänicke, Amélia Camarinha-Silva, Korinna Huber, Jana Frahm, Jana Seifert

**Affiliations:** 1 Institute of Animal Science, University of Hohenheim, Emil-Wolff-Str. 6-10, 70593 Stuttgart, Germany; 2 Institute of Animal Nutrition, Friedrich-Loeffler-Institute, Federal Research Institute for Animal Health, Bundesallee 37, 38116 Braunschweig, Germany

**Keywords:** Calves, Rumen, Weaning, Buccal swab, Stomach tubing, Non-invasive

## Abstract

**Background:**

The rumen bacterial communities are changing dynamically throughout the first year of calf’s life including the weaning period as a critical event. Rumen microbiome analysis is often limited to invasive rumen sampling procedures but the oral cavity of ruminants is expected to harbour rumen microbes due to regurgitation activity. The present study used buccal swab samples to define the rumen core microbiome and characterize the shifts in rumen and oral microbial communities occurring as result of calf’s age as well as time of weaning.

**Results:**

Buccal swab samples of 59 calves were collected along the first 140 days of life and compared to stomach tubing sample of the rumen at day 140. Animals were randomly divided into two weaning groups. Microbiota of saliva and rumen content was analysed by 16S rRNA gene amplicon sequencing. Our study showed that most rumen-specific bacterial taxa were equally observed in rumen samples as well as in the buccal swabs, though relative abundance varied. The occurrence of rumen-specific OTUs in buccal swab samples increased approximately 1.7 times from day 70 to day 140, indicating the gradual development of rumen as calf aged. The rumen-specific bacterial taxa diversity increased, and inter-animal variations decreased with age. Early weaning (7 weeks of age) rapidly increased the rumen microbial diversity from pre- to post-weaned state. Rumen microbiota of early-weaned calves seemed to have a suppressed growth of starch- and carbohydrate-utilizing bacteria and increased fibre degraders. Whereas, in late-weaned calves (17 weeks of age) no impact of dietary modifications on rumen microbiota composition was observed after weaning. Oral-specific bacterial community composition was significantly affected by calf’s age and time of weaning.

**Conclusions:**

The present study showed the significant impact of calf’s age and weaning on the establishment of rumen- and oral-specific bacterial communities utilizing buccal swab samples. The results emphasize the possibility of using buccal swab samples as a replacement of complex stomach tube method for large-scale predictive studies on ruminants. For in-depth rumen microbiome studies, the time of sampling should be carefully considered using an active phase of regurgitation.

**Supplementary Information:**

The online version contains supplementary material available at 10.1186/s42523-021-00095-3.

## Background

Dairy calves have an immature gastrointestinal tract (GIT) at birth, with a non-functional rumen [1]. The rumen proportions are relatively smaller than in adult cows and lack some major functional components (i.e., rumen wall villi), which are essential for nutrient absorption [1]. During the first 3 weeks of life, milk is the major component of diet, which is directly carried by an oesophageal groove into the abomasum without entering the rumen [1]. Therefore, the rumen contribution to nutrient digestion, absorption and generation of energy-rich substrates are marginal in young calves than in more advanced developmental stages.

The pre-weaning stage is a crucial period for the development of GIT and immune system in calves. The consumption of solid food as “starter feed” begins around second to third week of life. The highly palatable starter feed rich in rapidly fermentable carbohydrates triggers the growth and establishment of rumen microbiota, especially starch-degrading bacteria. The increase in fermentation products and microbial biomass result in structural and physiological modifications of rumen characteristics [2, 3], with subsequent establishment of a fully functional rumen and adult-like microbiota near weaning [4].

Several negative impacts of stressful weaning transition on animal feed intake and growth have previously been reported [5]. Weaning calves at 6 weeks of age rapidly shifted their rumen and faecal microbiome beta-diversity [6] and reduced their growth rate during weaning as compared to the late-weaned calves [7]. On the contrary, weaning calves at 8 weeks of age gradually shifted the beta-diversity, indicating a gradual increase in starter concentrate consumption and a progressive rumen development as compared to the early-weaned calves [6]. The premature weaning can increase the death rate in calves and delayed weaning could lead to increase feed cost and growth retardation of animal’s digestive organs, thereby, affecting productive performance of animals during maturity [8]. However, these effects can be minimized by careful consideration of weaning age of an animal, to ensure better intestinal and ruminal maturation before weaning [7].

The ruminal microbiome is non-static and changes continuously with diet [9, 10], host breed [11], age [12], as well as sampling time and location [13]. Moreover, inter-animal variations in rumen microbial community composition on a defined diet could be observed due to animal history, body condition and post-feeding sampling time, thereby, a larger sample size is needed to obtain ample statistical power. The majority of rumen sampling procedures in practice are invasive procedures such as rumenocentesis [14], oral intubation and rumen cannulation [15], which are not only unpleasant for the health and welfare of an animal but are also impractical for large scale animal sampling campaigns. Thus, the identification of more efficient, non-invasive, extensive rumen sampling procedure is needed for rumen microbiome studies.

Ruminants possess regurgitation activity that enables them to bring ruminal contents back to the mouth for chewing partially digested plant material [1]. Therefore, it is highly expected to obtain good representation of particle- and liquid-associated microbiota of the rumen utilising the buccal swab samples [16]. The concept of buccal fluid sampling as replacement of invasive rumen sampling procedures has already been tested in sheep fed on four different diet [16], and cows fed on grass silage-based diets with or without lipid supplementation [17]. However, for practical implication of the proof of concept, large scale non-invasive animal studies are needed. In the present study, buccal swab samples were collected using sterile cotton wool swabs at five different time points from 59 female Holstein calves weaned at 7 or 17 weeks of age. Bacterial communities of buccal swab samples were compared with rumen samples collected by stomach tubing from same animals at the end of experiment. The shifts in rumen and oral microbial communities occurring as a result of calf’s age as well as the time of weaning were also characterized.

## Results

### Characterization of feed intake pattern in relation to saliva sampling scheme

The microbial composition of buccal swabs is related to feed intake pattern, such as proportions of milk replacer (MR), concentrate feed (C) and roughage intake. The pattern of MR and C intake was similar for both weaning groups until day 28 when weaning was initiated for early-weaned (earlyC) group and there were no significant differences in intake amounts (122–133 g DM/day). While the late-weaned (lateC) group was maintained at a constant MR level of approximately 1300 g DM/day and continued to increase C intake until 2 kg was reached at day ~ 70, the MR intake of the earlyC group was gradually reduced until day 42. At this time, the C intake level of earlyC was similar to the lateC group. From day 42 onward, the earlyC group was fed a TMR. However, the intake level could not be recorded due to technical reasons. Saliva samples were taken at days 42, 70, 98, 112 and 140. Due to some technical and health-related issues, there was no access to all animals of the herd at each time point. In addition, some of the calves’ samples had to be removed during bioinformatic analysis due to poor sequence quality and low read counts. Thus, the final number of buccal swab (BS) samples used for the data analyses were: 11 (day 42), 26 (day 70), 51 (day 98), 51 (day 112), and 47 (day 140). Forty-seven samples were available from rumen (R) at day 140.

### Comparison of rumen and salivary bacterial communities of different age group calves

**Fig. 1:**
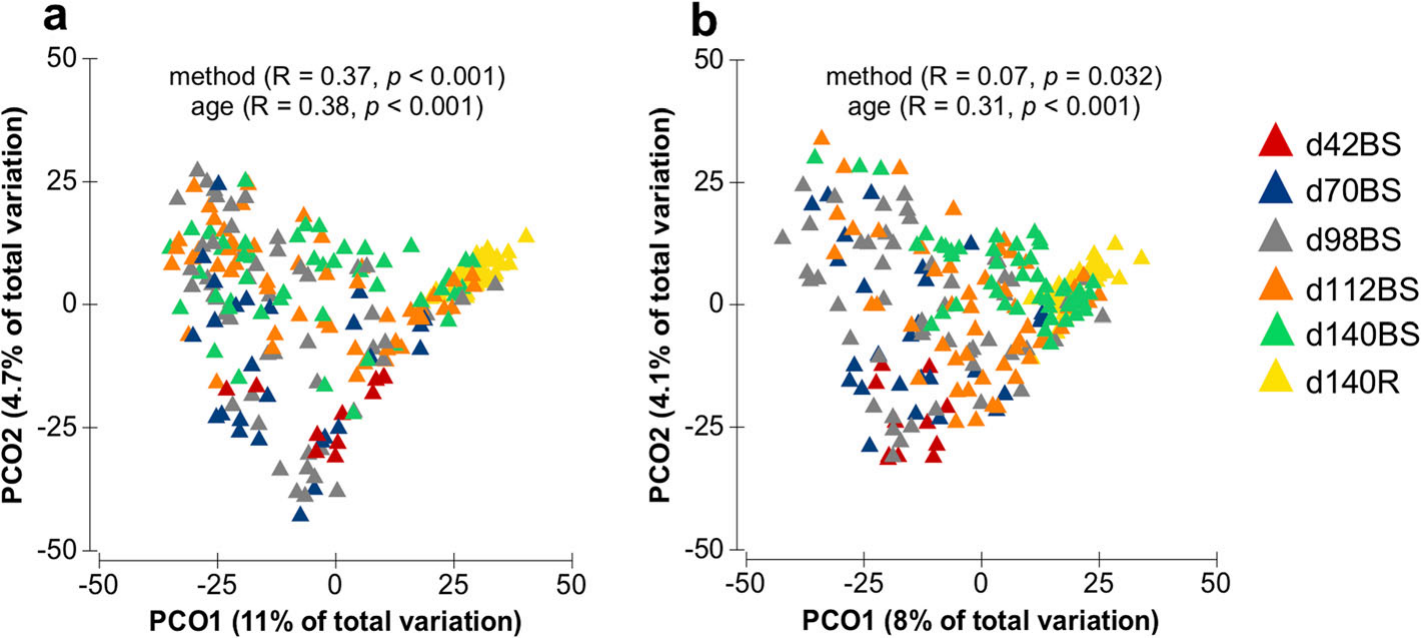
Principal coordinates analysis plots depicting the distribution of bacterial communities in 233 samples collected via two different sampling methods (buccal swabbing (BS) and stomach tubing (R)) from different age group calves, without exclusion (**a**) or after exclusion (**b**) of potential oral taxa by mathematical filtering approach. Each point represents one sample. Different age groups are indicated by different coloured triangles

Amplicon sequencing of BS and R samples revealed 17,716 ± 1590 mean read counts per sample for stomach tubing and 21,014 ± 2014 for BS samples and a total of 4906 unique bacterial operational taxonomic units (OTUs) were obtained. Bacterial communities in samples collected by stomach tubing clustered separately from those in samples collected via buccal swabs (Fig. 1a). Analysis of similarity revealed significant differences between sampling methods (stomach tubing vs. buccal swabbing; *p* < 0.001; *R* = 0.38; ANOSIM) as well as the calves age groups (*p* < 0.001; *R* = 0.37; ANOSIM). This was due to high relative abundances of potential oral bacterial taxa in the BS samples. Exclusion of OTUs related to oral bacteria was done by using a previously described mathematical filtering approach [16]. According to this approach, all the bacterial genus-level taxa with maximum relative abundance of ≥1% (arbitrary cut-off) in BS samples as compared to any sample collected by stomach tubing were classified as “true” oral bacteria. This resulted in an oral-specific (OS) dataset of 1190 OTUs corresponding to 141 genera as potential oral bacteria. The rumen-specific (RS) dataset included 3479 OTUs, where 29 genus-level taxa were grouped as potential rumen bacteria. The OS-taxa excluded with the mathematical filtering approach made up 36.0, 66.2, 65.0, 53.2 and 57.1% of the total bacterial communities of the day 42, 70, 98, 112 and 140 BS samples, respectively. In addition, the bacterial taxa with maximum relative abundance of < 1% in all of the BS and R samples (237 OTUs corresponding to 104 genus-level taxa) were classified as rare taxa (Additional file 2: Table S8.1 and S8.2). The rare taxon accounted for a maximum of 0.01–0.97% contribution to the total bacterial community across all samples, thus considered not important for the study and eliminated from further analysis.

Following normalization of the 29 potential RS genus-level taxa to account for a total of 100% in each sample, analysis of beta-diversity revealed better clustering of samples by calves age groups (*p* < 0.001; *R* = 0.31; ANOSIM) rather than by the sampling method used (*p* = 0.032; *R* = 0.07; ANOSIM) (Fig. 1b).

### Effect of calves age on rumen-specific microbiota

The effect of calves age on RS microbiota was analysed without taking into consideration the time of weaning. There was a significant effect of calves age (*p* < 0.01) on RS bacterial diversity, as indicated by a significant gradual increase in alpha-diversity from 3.91 (day 42BS) to 4.27, 4.36 and 4.50 at days 98, 112 and 140 BS samples, respectively (Additional file 1: Figure S1). In addition, the inter-animal variations decreased with calves age as indicated by a lower spread of Bray-Curtis values in older animals (Fig. 1b). An increased within-group similarity from 9.6% at day 70 to 18.9% at day 140 (Additional file 1: Figure S7), as well as higher number of shared RS-OTUs with animal age (Additional file 1: Figure S2) were observed. However, exception was observed for day 42 BS samples, which showed a higher within group similarity (15.0%) compared to the BS samples of all other time points. This was probably due to the low sample number (*n* = 8) compared to the other time points (*n* = 24–48) as well as the influence of feed intake. At this time point, milk replacer (MR) intake in earlyC animals was low, whereas the C intake level of earlyC was similar to the lateC group. Thus, the overall feed composition was similar consisting predominantly of MR and C. However, at days 70 and 98, the two weaning groups clearly had different dietary conditions. While, at day 112 and 140, both weaning groups started to receive the comparable dietary ration, thus, resulting in increased within-group similarity in older animals.

Calves age significantly modified the RS bacterial community composition as indicated by a decrease in relative abundance of phylum *Actinobacteria* (*p* < 0.001) and an increase in *Bacteroidetes* (*p* < 0.001), *candidatus Saccharibacteria* (*p* < 0.001), *Fibrobacteres* (*p* < 0.019), *Proteobacteria* (*p* < 0.015), and *SR1* (*p* < 0.001) with age of calves (Additional file 1: Figure S3a, Additional file 2: Table S1). At genus-level, a continuous significant decrease in relative abundances of genera *Olsenella*, unclassified *Prevotellaceae,* unclassified *Lachnospiraceae*, and a subsequent significant increase in unclassified *Bacteroidetes*, unclassified *Bacteroidales*, unclassified *Saccharibacteria genera incertae sedis*, *Fibrobacter*, *Ruminobacter*, and unclassified *SR1 genera incertae sedis* from days 42–140 was observed (Additional file 1: Figure S3b, Additional file 2: Table S1).

In addition to the calves age, sampling method also significantly affected the RS bacterial community composition as indicated by high relative abundances of genera: unclassified *Saccharibacteria genera incertae sedis*, unclassified *Clostridiales*, unclassified *Ruminococcaceae,* unclassified *SR1 genera incertae sedis,* and lower relative abundance of unclassified *Prevotellaceae* in rumen samples as compared to the BS (Additional file 1: Figure S3b, Additional file 2: Table S1).

The developing calves rumen core microbiome was defined at the described circumstances of housing and feeding conditions based on BS samples collected from 70 to 140-day-old calves (irrespective of weaning time), as the microbiota of 6–12-week-old calves resembled more closely to the adult-like microbiota rather than early developmental stages [18]. A total of 3425 unique RS-OTUs were defined in this time period showing varying numbers at the single time points. The occurrence of RS OTUs in BS samples increased with age of calves from 726 OTUs (day 70) to 1243 OTUs (day 140), indicating the gradual development of rumen and its microbiome. Out of this, 614 RS-OTUs were defined as “core bacterial OTUs” commonly found in day 70BS, day 98BS, day 112BS, day 140BS and day 140R samples (Additional file 1: Figure S2). These core OTUs were taxonomically associated to 8 bacterial phyla and 27 genus-level taxa, with 331 OTUs assigned to *Bacteroidetes*, 196 OTUs to *Firmicutes*, 19 OTUs to *Actinobacteria*, 14 OTUs to *candidatus Saccharibacteria*, 10 OTUs to *Proteobacteria*, 9 OTUs to *Fibrobacteres*, 7 OTUs to *SR1*, 2 OTUs to *Tenericutes* and 26 OTUs were assigned to an unknown bacterial phylum (Additional file 2: Table S2).

**Fig. 2:**
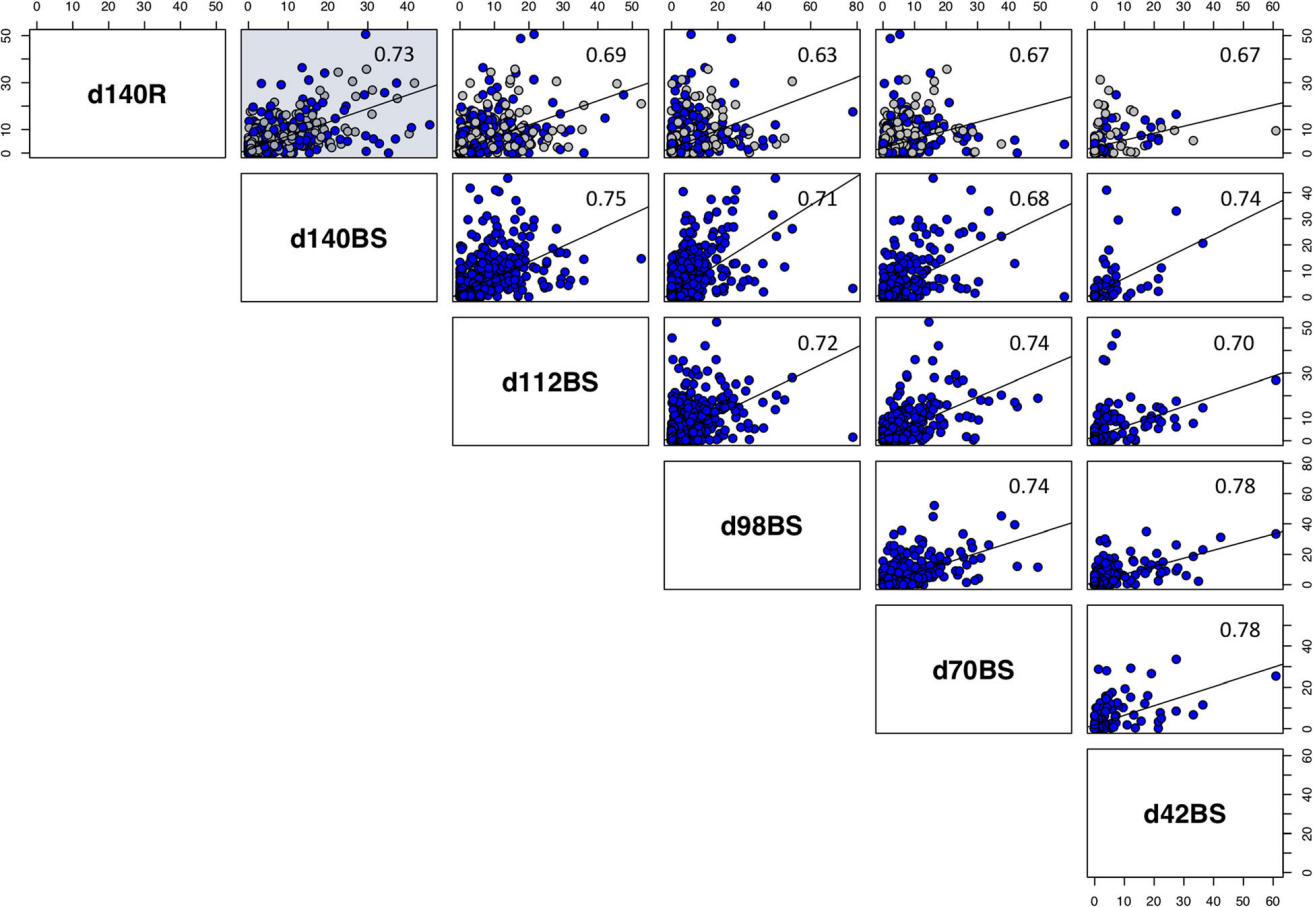
Scatter plots for analysis of differences in the relative abundances of each RS bacterial taxon, among sample types. The circles (o) are representing RS bacterial taxon in R and BS samples. The same animals were compared among sample types: a total of 36 animals (day 140R vs. day 140BS), 36 animals (day 140R vs. day 112BS), 35 animals (day 140R vs. day 98BS), 19 animals (day 140R vs. day 70BS) and 6 animals (day 140R vs. day 42BS). Spearman correlation coefficients (R-values) are indicated. Correlation in terms of microbial taxa abundance between d140R vs. d140BS is illustrated in the upper left corner highlighted with a light blue background. Grey dots represent RS samples and blue dots represent BS samples

In scatter plots, the relative abundances of 29 RS bacterial genus-level taxa from BS samples of five age group calves (days 42, 70, 98, 112 and 140) were compared individually with day 140 rumen samples and the strength of similarity among sample types was assessed based on overall Spearman correlation coefficient (Fig. 2). BS samples collected from 140-day-old calves were most similar (*R* = 0.73), while the ones collected from other age group calves were less similar (*R* = 0.63–0.69) with the rumen bacterial composition. In addition, an overall high correspondence was observed between all BS samples regardless of calves age, with R-value ranging between 0.68–0.78. The extent to which the BS samples collected at day 140 reflected the rumen microbial community composition at the same time point was assessed using a Mantel test. The BS Bray-Curtis dissimilarity matrix had a significant relationship with the rumen Bray-Curtis dissimilarity matrix (Mantel statistic *R* = 0.28, *p* < 0.001) meaning that samples which became more dissimilar in terms of RS microbial community composition in BS samples also became more dissimilar in terms of microbial community composition in R (Additional file 2: Table S9). The fitness of the BS-RS approach in reflecting the rumen microbiome composition was further elucidated based on Spearman correlation coefficient between the OTUs relative abundance along the d140R samples with its abundance over the RS portion of the d140BS samples yielding an average R-value of 0.21 (Additional file 1: Figure S8).

### Effect of weaning time on rumen-specific microbiota in calves

**Fig. 3:**
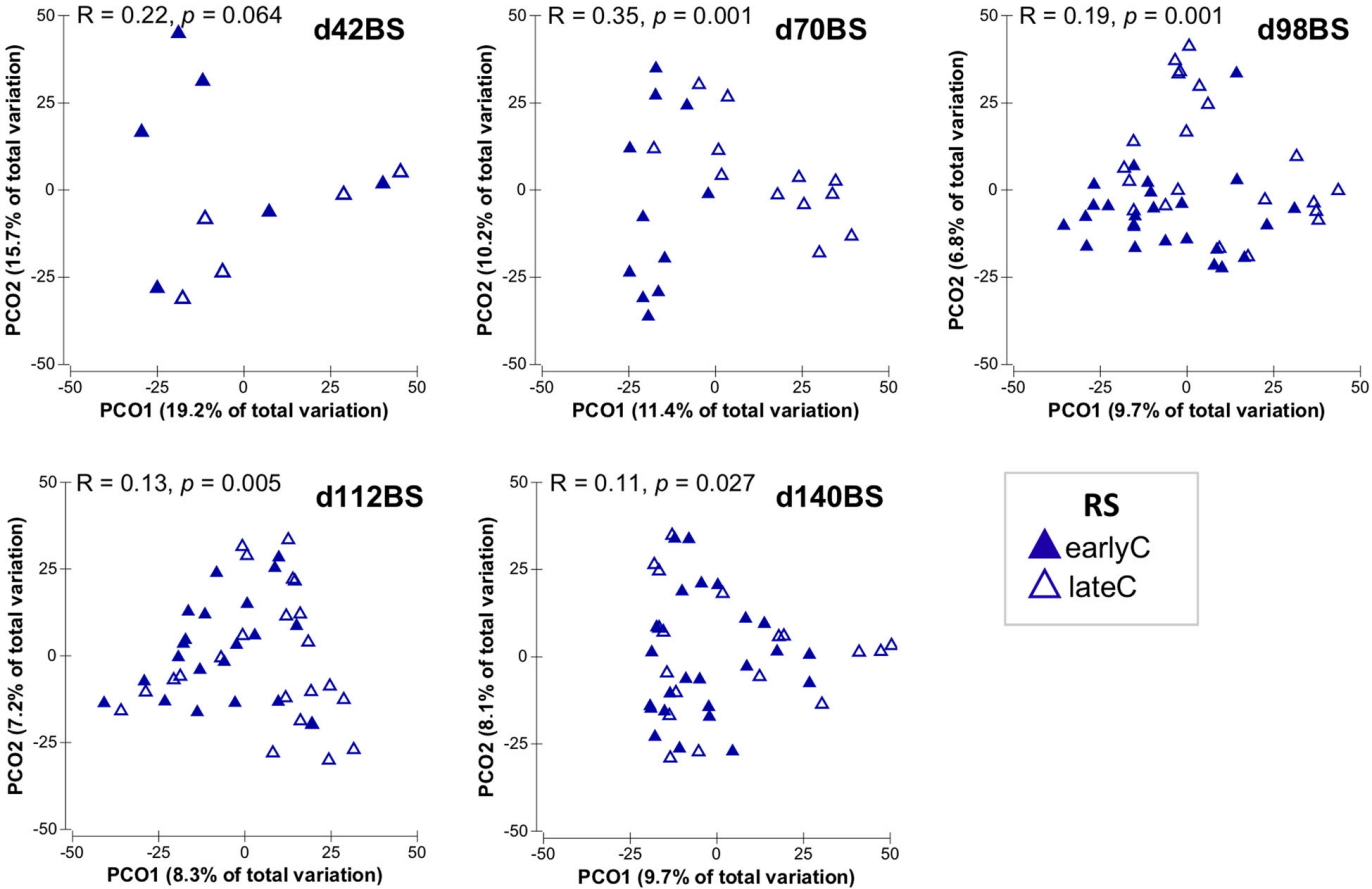
Principal coordinates analysis plots depicting RS bacterial communities in BS samples of earlyC and lateC groups. Each triangle represents one sample

**Fig. 4:**
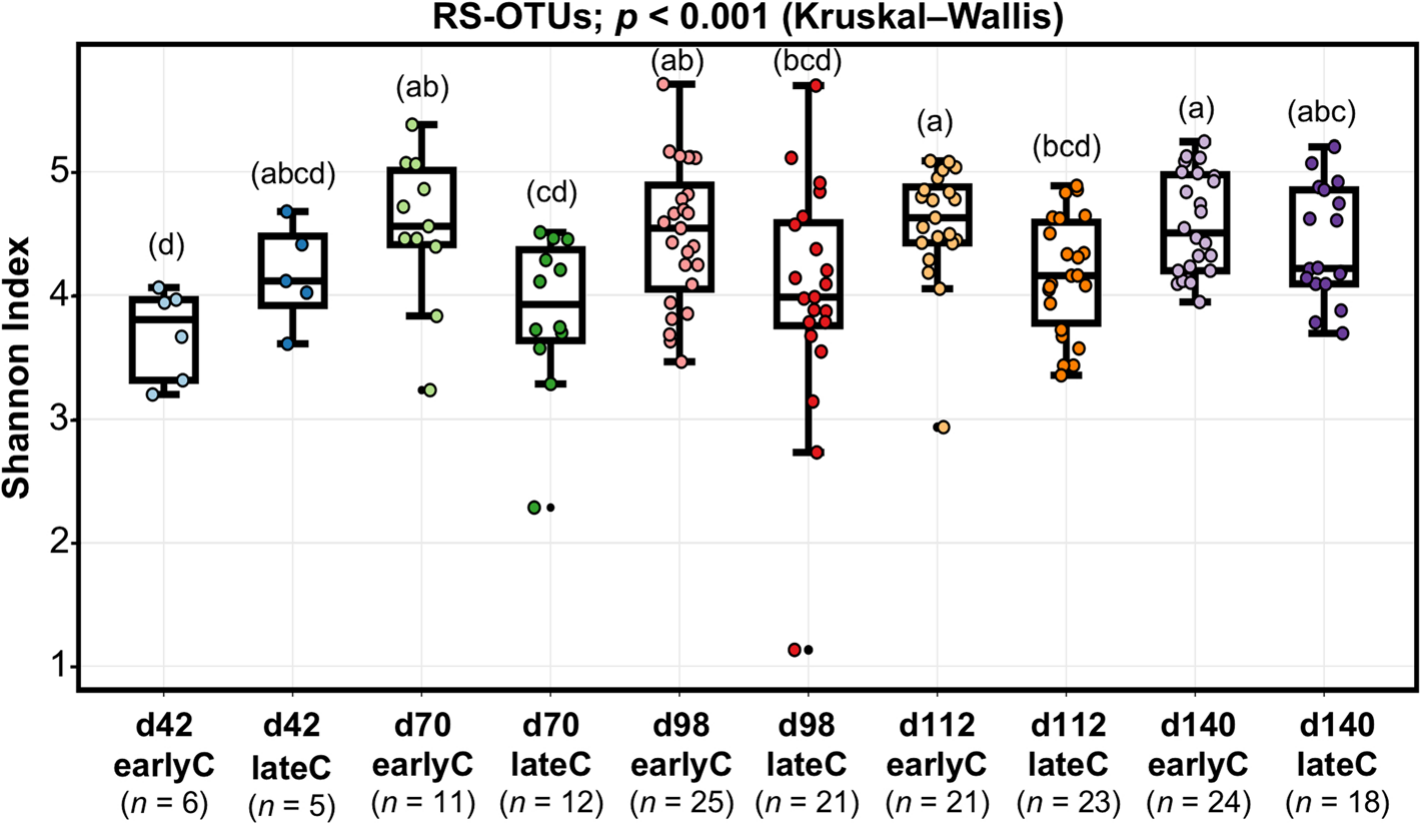
Shannon index of RS bacterial communities in BS samples of different weaning groups of calves. Different weaning periods within each age group are shown by different colours

In addition to the calves age, the RS bacterial communities in BS samples were also affected by the time of weaning, as indicated by separate clustering of RS bacterial communities of earlyC and lateC groups specifically during days 70 and 98 (Fig. 3). Analysis of similarity (ANOSIM) revealed significant differences between weaning groups at days 70, 98 and 112 (Additional file 2: Table S3). Significant differences were also observed in bacterial diversity (Shannon index; *p* < 0.001). EarlyC had higher taxa diversity with a rapid increase from pre- to post-weaning period compared to the lateC group. However, the lateC group only showed a gradual increase in taxa diversity with calves age without any prominent impact of weaning (Fig. 4).

**Fig. 5:**
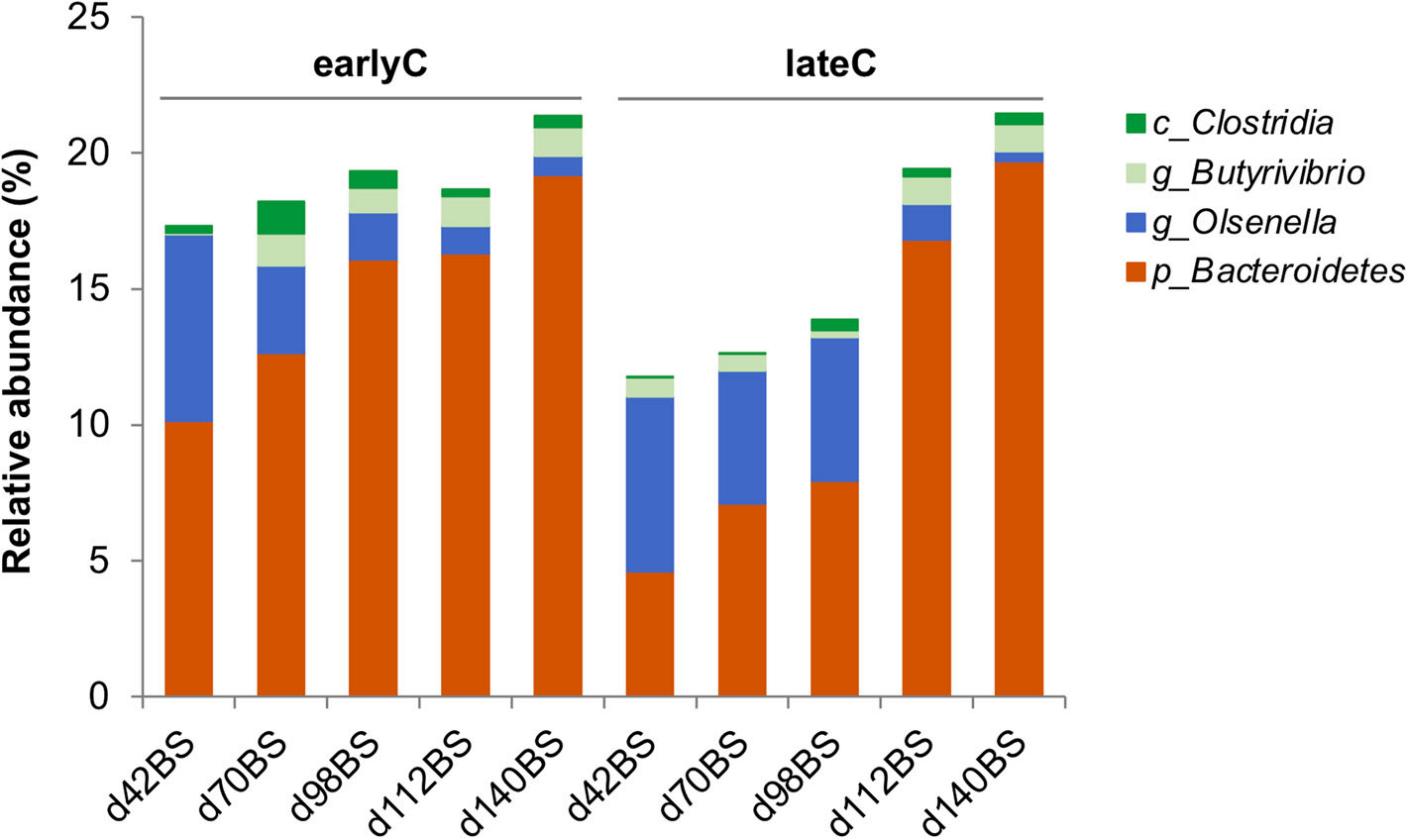
Average relative abundances of RS bacterial genus-level taxa in BS samples of different weaning groups of calves. Each bar represents an average value for animals at each age group-weaning period combinations: day 42BS (6 & 5 animals), day 70BS (11 & 12 animals), day 98BS (25 & 21 animals), day 112BS (21 & 23 animals) and day 140BS (24 & 18 animals) for earlyC and lateC groups respectively. Only taxa that showed significant differences (*p* ≤ 0.05) between the two weaning groups in a given sampling day are indicated

Comparing the RS bacterial community composition of earlyC group with the same day-old lateC group, no significant effects of weaning time were observed at the phylum-level (*p* > 0.05) (Additional file 2: Table S4). Nevertheless, at the genus-level, earlyC group showed significant higher relative abundance of genus unclassified *Clostridia* (*p* = 0.002) at day 70 as compared to the same day-old lateC group. In contrast, lateC group showed a significant higher relative abundance of genus *Olsenella* (*p* < 0.001) and lower relative abundances of unclassified *Bacteroidetes* and *Butyrivibrio* at day 98 as compared to the same day-old earlyC group. No significant differences were observed between RS bacterial communities of weaning groups at days 42, 112 and 140 (Fig. 5, Additional file 2: Table S4).

### Effects of calves age and time of weaning on oral-specific microbiota

The effects of calves age and time of weaning on OS microbiota were analysed separately. Following normalization of the 141 potential OS genus-level taxa to account for a total of 100% in each sample, analysis of beta-diversity revealed minor age-related clustering of the OS bacterial communities of calves (*p* = 0.001; *R* = 0.11; ANOSIM) (Additional file 1: Figure S4A). At phylum-level, a significant decrease in relative abundance of *Fusobacteria* (*p* = 0.014) and a subsequent significant increase in *Proteobacteria* (*p* = 0.048) with age of calves was observed (Additional file 1: Figure S4b, Additional file 2: Table S5). At genus-level, an age-dependent decrease in relative abundances of unclassified *Flavobacteriaceae*, unclassified *Porphyromonadaceae, Corynebacterium, Acidaminococcus, Roseburia*, *Anaerostipes, Bacteroides*, *Actinomyces, Selenomonas, Blautia*, and *Ruminococcus 2* were observed. Some of these genera (*Bacteroides, Actinomyces, Selenomonas, Blautia,* and *Ruminococcus 2*) were highly abundant at day 42 as compared to the BS samples of other time points and it was probably not related to calf’s age or weaning rather an effect of low sample number at this specific time point. In addition, few OS genera also showed increased abundances with age such as *Acinetobacter*, *Burkholderia, Rhizobium, Arthrobacter*, *Aerococcus, Variovorax* and *Clavibacter* (Additional file 1: Figure S5, Additional file 2: Table S5).

**Fig. 6:**
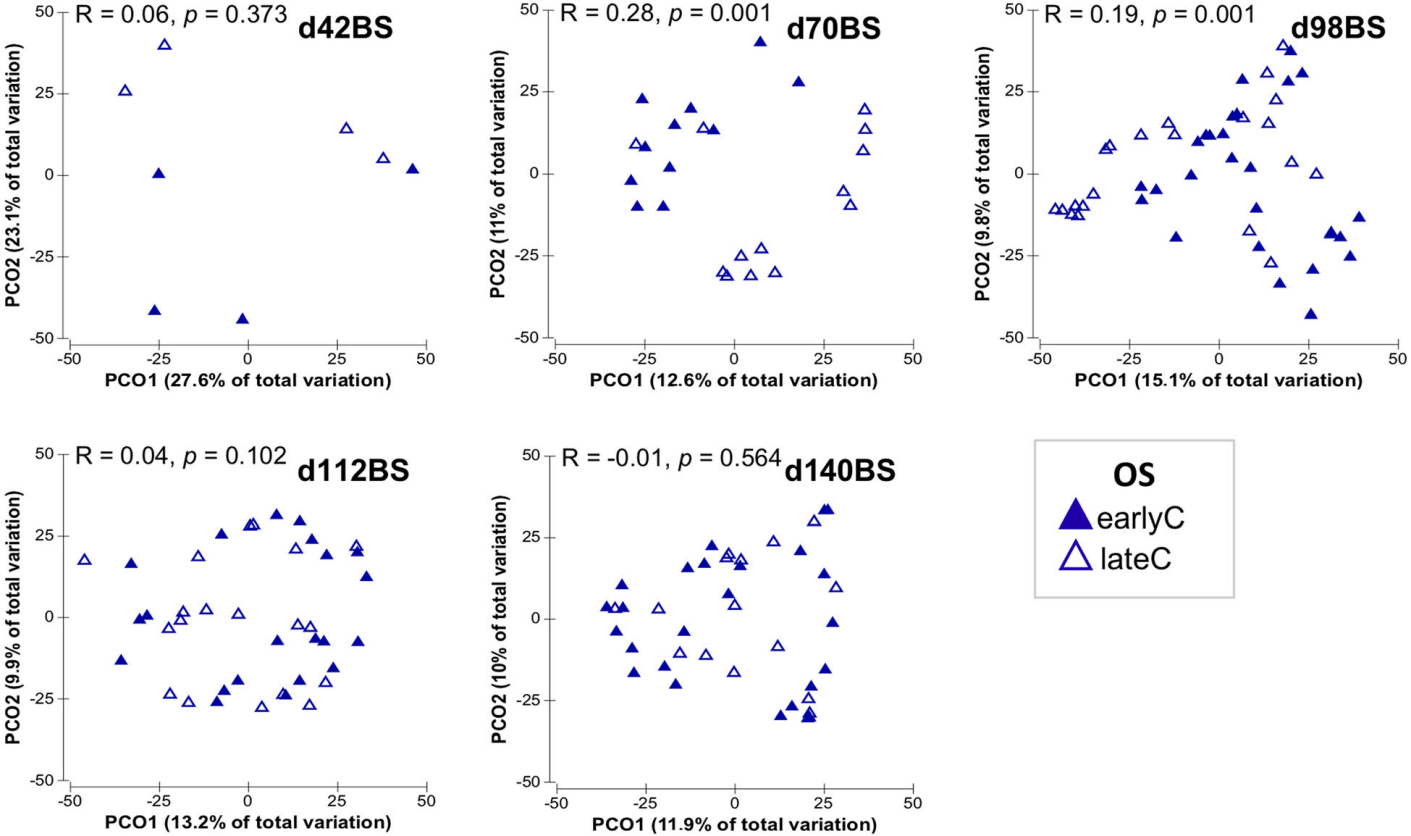
Principal coordinates analysis plots of OS bacterial communities in BS samples of earlyC and lateC groups. Each triangle represents one sample

**Fig. 7:**
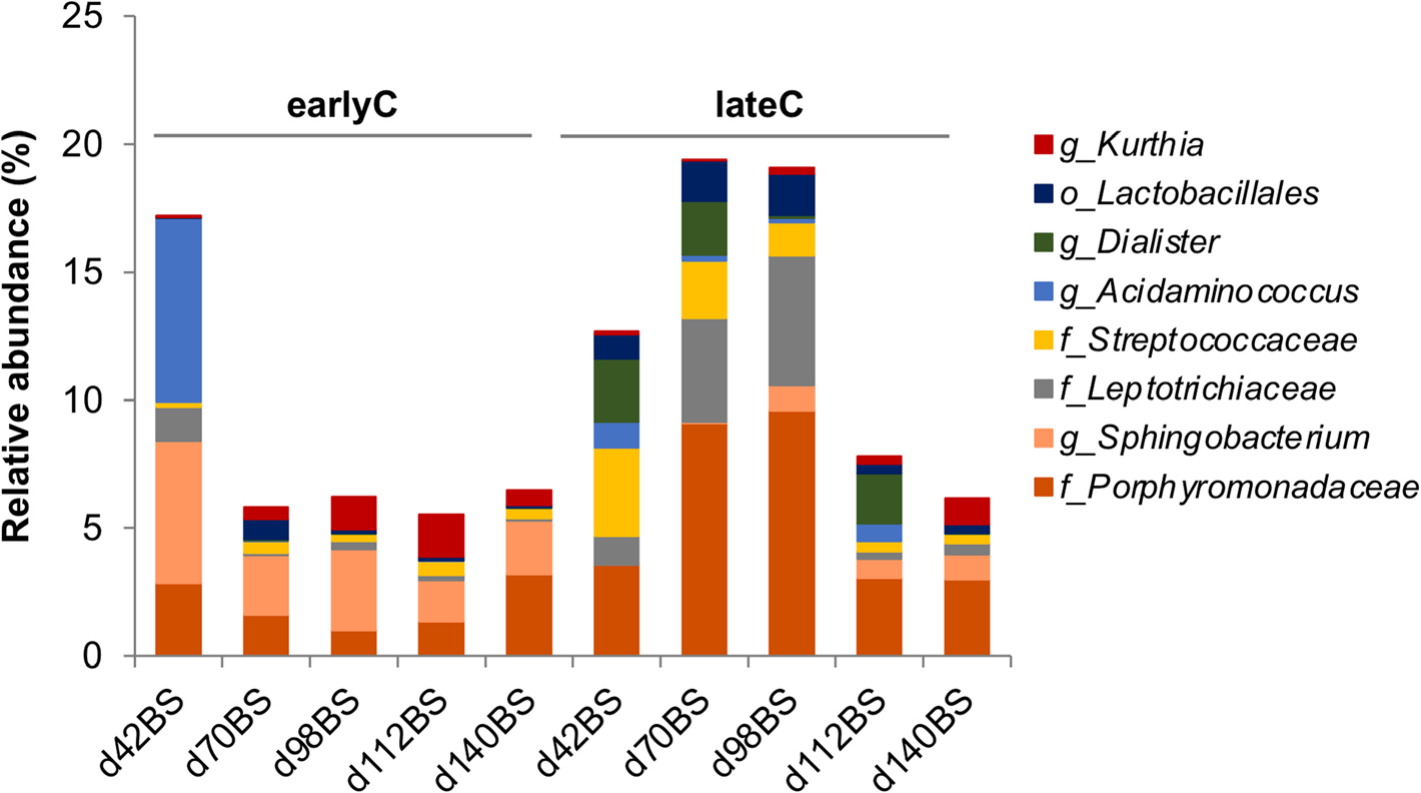
Average relative abundances of OS bacterial genus-level taxa in BS samples of different weaning groups of calves. Each bar represents an average value for animals at each age group-weaning period combinations: day 42BS (4 & 4 animals), day 70BS (11 & 13 animals), day 98BS (27 & 21 animals), day 112BS (21 & 20 animals) and day 140BS (24 & 16 animals) for earlyC and lateC groups respectively. Only taxa that showed significant differences (*p* ≤ 0.05) between the two weaning groups in a given sampling day are indicated

Weaning affected the OS microbiome mainly at days 70 and 98 of calf’s life, as indicated by separate clustering of OS bacterial communities of earlyC and lateC groups during respective days (Fig. 6, Additional file 2: Table S3). Both calves age and time of weaning were found to have no significant impact on OS bacterial diversity (Shannon index; *p* > 0.05, Additional file 1: Figure S6). Comparing the OS bacterial community composition of the two weaning groups, earlyC group showed a significant decrease in relative abundance of phylum *Fusobacteria* at day 98 as compared to same day-old lateC group and the relative compositions of other OS-phyla remained unaffected by the time of weaning (Additional file 2: Table S6). Nevertheless, weaning time clearly influenced the OS bacterial community composition at the genus-level, where the earlyC group had significantly higher abundances of genera *Sphingobacterium* (days 42–70), *Kurthia* (day 70), and lower abundances of genera *Dialister* (day 42), *Acidaminococcus* (day 70), unclassified *Lactobacillales* (day 98), unclassified *Porphyromonadaceae* and unclassified *Leptotrichiaceae* (days 70–98), and unclassified *Streptococcaceae* (days 42–98) as compared to the same day-old lateC group. No significant differences were observed between OS bacterial communities of weaning groups at days 112 and 140 (Fig. 7, Additional file 2: Table S6).

## Discussion

The progressive development of rumen to mature state occurs with age and the transition phase at weaning [6]. The start of solid food intake initiates ruminal fermentation processes which greatly modifies the rumen microbial community composition. In the present study, we defined the rumen core microbiome using BS samples and then characterized the shifts in rumen and oral microbial communities occurring as result of calf’s age as well as the time of weaning. Obtaining rumen samples via stomach tubing is usually a laborious and technically challenging procedure [19]. It is a stressful event for the animals and can have negative impacts on animal health. Therefore, rumen fluid samples from the young calves (days 42, 70, 98 and 112) were not collected in our study. On the contrary, collection of BS samples from oral cavity of an animal is a less time consuming, non-invasive method and can possibly be used as a replacement of complex stomach tube method to study rumen microbiota [16, 17]. Many previous studies characterized the rumen microbial communities of pre-ruminant calves using sacrificed animals, with the limitation of long-term monitoring of single animal [18, 20–22]. This can be circumvented with the use of BS, as it replaces the need to sacrifice the animals and enables the monitoring of animals across their entire life span, without having any harmful impact on animal’s health due to repeated non-invasive sampling procedures. In the present study, the major RS bacterial taxa observed in the stomach tube samples were also detected in the BS samples, though relative abundances varied. Exceptions were *Anaeroplasma, Fibrobacter, Ruminobacter,* unclassified *candidatus Saccharibacteria* and unclassified *Elusimicrobiales* that were absent in the BS samples of 42-day-old calves, which is in agreement with other studies that reported these bacterial taxa to be very low abundant or totally absent in the rumen of 2 months old calves [12, 23]. Moreover, the occurrence of RS-OTUs in BS samples increased approximately 1.7 times from day 70 to day 140 independent of the time of weaning, indicating the gradual development of rumen as calf aged. In general, the BS samples of 140-day-old calves showed high overall correspondence and similar bacterial taxa diversity to the stomach tubing samples collected at the same day. Besides the valid representation of rumen microbiota, OS bacterial taxa were also identified. As the time passes between the regurgitation activity and sampling, the amount of typical oral bacteria increases in the buccal swabs [16]. Therefore, the dataset was filtered to remove these oral bacterial taxa using a mathematical filtering approach [16], that resulted in a clear clustering of bacterial communities by calf’s age or time of weaning rather than by sampling method used.

In the present study, hay (ad libitum) and concentrate feed (max. 2 kg/day) were available for the calves throughout the experimental period [24], thus it could be suggested that rumen fermentation processes have already started prior to weaning. Calf’s age affected the diversity of RS microbiome as shown by an increase in taxa diversity and a decrease in inter-animal variation with age. Our results are in agreement with a recent study on age-dependent shifts in gut communities of dairy cows, showing lower beta-diversity and a higher alpha-diversity with age [18].

Weaning also affected the bacterial diversity in the rumen. Early weaning (7 weeks of age) rapidly increased the microbial diversity from pre- to post-weaned state (days 42–70) proven by a similar diversity in more mature age (days 98–140). Such an abrupt shift in microbial diversity of earlyC group reflects the sudden alteration in the source of nutrients for calves, paralleled by an overall reduced growth of earlyC compared to the lateC group [24]. A Spearman correlation analysis also showed strong positive correlations between bacterial alpha-diversity and body length (*R* = 0.58; *P* = 0.048) only during day 70 in lateC group (Additional file 2: Table S7). Conversely, lateC group (17 weeks of age) showed a gradual increase in microbial diversity with age rather than weaning, indicating age-dependent gradual increase in intake of concentrate [24], and perhaps progressive development of rumen as compared to the earlyC group. Overall, lateC group showed lower microbial diversity than earlyC from day 70 to day 112 and this was perhaps due to consumption of high amount of concentrate feed (starch) in lateC group prior to weaning. A reduction in rumen bacterial diversity was previously observed with dietary starch addition in Holstein cows [25] and was suggested to be linked to an improved feed efficiency in dairy cows [26].

Changes in diversity were correlated to phylogenetic modifications. The relative abundance of phylum *Actinobacteria* decreased, while *Bacteroidetes*, *candidatus Saccharibacteria*, *Fibrobacteres*, *Proteobacteria*, and *SR1* increased with age of calves. However, the time of weaning did not modify the rumen bacterial composition at the phylum-level. The genus-level composition showed that the dominant genera belonging to *Actinobacteria* namely *Olsenella* was affected by both calves age as well as time of weaning, as indicated by a significant decrease in its relative abundance with age and lower relative abundance in earlyC group as compared to the lateC group. *Actinobacteria* were described to be dominant in newborn calves exclusively fed with colostrum and showed an age-dependent decrease and compositional change in older animals [12]. This verified their role as early colonizers of neonate’s gut and their importance for the conversion of milk components. In addition, they are related to the consumption of starch to produce lactic acid [27] and *Olsenella* ferments carbohydrates to produce lactic, formic and acetic acid [28]. A decrease in *Olsenella* abundance with dietary forage inclusion was recently reported [29]. Thus, it can be speculated that the decrease in *Olsenella* abundance with age and after day 70 in the earlyC group was probably due to weaning related dietary modifications as milk replacer was substituted by a total mixed ration (TMR) including 48% grass silage.

*Bacteroidetes* showed an increase in abundance with age and after weaning events in the present study. The main reason was the change in diet and the corresponding availability of plant polysaccharides which could be used by *Prevotella* spp. and other members of the *Bacteroidetes* phylum, inventing a huge number of carbohydrate active enzymes [9]. Changes within the *Firmicutes* phylum were especially observed in the increased abundance of unclassified *Clostridia* after weaning (day 70 earlyC). The high abundance of unclassified *Clostridia* in post-weaned microbiota of earlyC group was diet-dependent, as high abundances of *Clostridia* have previously been reported using diets containing forages and mixed forages [30]. In addition, an age-dependent increase in the abundance of *Fibrobacteres* and it corresponding *Fibrobacter* genus was observed. *Fibrobacteres* are major degraders of cellulose in the rumen [31] and their abundance in rumen decreases with increasing dietary concentrate proportions [32]. Thus, the increased abundance of *Fibrobacteres* in the rumen of mature calves in our study seemed to be reasonable due to the presence of hay and a total mixed ration in their diet.

The calves age as well as the time of weaning also affected the OS microbiota at both phylum- and genus-level. A recent study reported that the oral microbiota of neonatal calves matured quickly and contained similar microbial composition to the adult cow oral microbiota by four-weeks of age [33]. In the present study, the oral samples were collected at seven-weeks of age, thus, it is presumable that the oral microbiota was matured and the major changes observed in OS microbiota composition were mainly caused by weaning related dietary shifts. Weaning influenced the OS microbiota mainly at days 70 and 98 of the calf’s life, where the earlyC group showed significant higher abundances of genera *Kurthia* and *Sphingobacterium*, and lower abundances of *Dialister*, *Acidaminococcus*, and unclassified *Lactobacillales* as compared to the same day-old lateC group. *Kurthia* occupied the normal intestinal microbiota of high-roughage fed cattle [34]. *Dialister* was frequently isolated from the oral cavity, with some species as causative agents of periodontitis [35]. This genus was positively correlated with starch degradation [36] and the decreased abundance of this genus in earlyC group after weaning was due to weaning related dietary shifts. *Lactobacilli* are common members in GIT of human and animals, in mouth and female genital tract and exert certain beneficial effect on host health such as reduced diarrhoea and increased weight gain in neonatal calves, provide protection against pathogenic bacteria, promote gut health and reduce gastrointestinal inflammatory responses [37]. *Lactobacilli* concentration was high in milk consuming calves [2] and negatively affected by weaning [38]. The higher abundance of *Lactobacilli* observed in 98-day-old lateC group in our study was probably due to higher availability of rapidly fermentable substrates (e.g., starch and lactose) in their diet compared to the earlyC group, receiving a total mixed ration.

The present data showed an age-dependent decrease in potential pathogenic bacteria such as *Corynebacterium,* unclassified *Flavobacteriaceae* and unclassified *Porphyromonadaceae*. *Corynebacterium* colonizing the skin and membranes in animal and humans [39], including several disease-causing species such as *C. bovis*, a causative agent of bovine mastitis [40]. *Flavobacteriaceae* family members were found in human oral cavity, dog mouth and other habitats [41, 42]. Certain *Flavobacteriaceae* genera (*Flavobacterium* and *Bergeyella*) can cause dental caries [43]. Similarly, the weaning related dietary shifts also benefited the post-weaned microbiota of earlyC group in terms of decreased abundances of potential pathogenic bacteria such as *Porphyromonadaceae* and *Leptotrichiaceae*. Species belonging to *Porphyromonadaceae* are ubiquitously present in oral cavities and GIT of animals and humans with some causing infections [44]. Genera belonging to *Leptotrichiaceae* such as *Leptotrichia* are commonly found in human oral cavity and are causative agents of dental plague [45]. In addition, no significant differences were observed between RS as well as OS bacterial communities of weaning groups at days 112 and 140, indicating greater ruminal maturation and enhanced feed adaptability of calves’ microbiota at 17 weeks as compared to 7 weeks of age.

## Conclusion

Our study showed the significant impact of calves age and time of weaning on the establishment of RS and OS bacterial communities using BS samples. This sampling strategy eliminated the need of animal slaughtering or invasive rumen sampling and enabled sample collection from a large number of animals over a longer time span. The results of our study are emphasizing the possibility of using BS samples in large-scale predictive studies on ruminants, where direct access to the ruminal contents is not an option. However, the BS dataset should be carefully evaluated, when analysing the abundances of RS microbiome. The oral health of an animal and the gap between the regurgitation activity and sampling can increase the amount of typical oral or pathogenic bacteria in the buccal swabs decreasing the predictive power of buccal swabbing procedure. In addition, sampling via buccal swabs might serve as a potential tool for the establishment of a fast-screening methodology to monitor the weaning status of calves. Prospective lab-on-chip techniques using probes specific for OS and RS taxa could be developed to provide an easy-to-use diagnostic tool for the farmers and to avoid illegal calf trading. This study identified 614 “core RS bacterial OTUs” corresponding to 27 genus-level taxa that were ubiquitously observed in BS samples of 70–140-day-old calves. The obtained dataset might serve as starting point to define potential biomarker OTUs in future predictive studies on ruminants. In addition, our study exposed the beneficial effects of late weaning in terms of relatively stable rumen and oral microbial community composition, quick adaptability of microbiota to dietary changes and better growth performance in lateC group.

## Methods

### Animals management and diets

The experimental design was previously described by Schwarzkopf et al. [24]. Briefly, 59 female German Holstein calves born to an established herd in a seasonal calving period (October to December) were monitored from birth until 149 ± 2 days of life. Calves were fed initially after birth with 3 L of colostrum using nipple bucket. Within 2–3 h after birth, calves were shifted into straw-bedded single hutches and were fed twice a day with 2 L of pooled herd milk. During the pre-experimental period (starting from 3 days of age), pooled herd milk was mixed with milk replacer (MR; NOLAC GmbH, Zeven, Germany). The milk replacer was first dissolved in temperature-adjusted water and then mixed with pooled herd milk. The amount of MR was increased gradually from 0.3 kg/d (day 3) to 0.9 kg/d (day 5), with a maximum amount of liquid feed available at a concentration of 150 g/L MR. The experimental period started by shifting calves at an average age (8 ± 1.9 days) and live weight (44.5 ± 5.2 kg) into two separate, straw-bedded free barns within one housing facility and animals were kept randomized in the groups until weaning. Both compartments of the barn were under the same climatic conditions and equipped with MR and concentrate self-feeding stations (Förster-Technik GmbH, Engen, Germany). Each calf was equipped with an ear transponder for automatic recording of the daily individual intake of MR and concentrate. During the first 5 days of experimental period, both groups received 0.9 kg MR powder/d. MR amount was gradually increased from 0.9 kg/d (day 6) to 1.35 kg/d (day 10) and remained at constant level until start of weaning. Over the entire experimental period, the maximum amount of liquid feed was available at a concentration of 150 g/L MR, water was fed ad libitum and a maximum amount of 2 kg concentrate feed/d was available until weaning. At the time of weaning, calves were moved to another straw-bedded barn in groups of different sizes. EarlyC group was weaned at 7 weeks of age (days 28–42) and lateC group at 17 weeks of age (days 98–112). During weaning, the amount of milk replacer was reduced in a 14 days step-down approach from 1.35 kg/d to 0.3 kg/d. Concentrate amount was reduced from 2 kg/d to 1 kg/d during weaning at day 98 for lateC animals. Reducing the concentrate in lateC group was intended to reduce the risk of acidification of the rumen during weaning and to stimulate roughage intake and consequently rumen development at the same time. After weaning all calves were housed irrespective of their weaning group affiliation together into two compartments within one straw-bedded barn under same housing conditions and were fed ad libitum with hay and a total mixed ration comprising of grass silage (48%), maize silage (32%) and concentrate feed (20%). The individual concentrate intake until weaning was previously published by Schwarzkopf et al. [24].

### Sampling procedures

#### Buccal swabbing using sterile cotton wool swabs

On experimental days, buccal swabs were taken in the morning between 8.00 and 11.00 am. Since the calves had no fixed feeding times, sampling was independent of the feeding time. Two sterile cotton wool swabs were placed on a clamp in the mouth of each calve for at least 30 s, at day 42, 70, 98, 112, and 140 of the experimental periods. Cotton wool swabs were immediately inserted into salivette (Sarstedt, Nümbrecht, Germany) and cooled on ice. The salivettes were centrifuged at 2000 g for 3 min and the swabs were frozen individually in plastic bags at − 80 °C. The bacterial cells were eluted from the BS samples by mixing them with 4 mL PBS buffer, incubated in fridge for 1 h, followed by 30 s sonication using ultrasonication bath. The liquid was squeezed from the swabs with sterile forceps. The extracted sample was centrifuged at 2500 g for 10 min, supernatant was transferred into clean tubes and centrifuged again at 19,000 g for 10 min. Half of the supernatant was discarded, and pellet was resuspended in the remaining supernatant. After 15 s sonication step, the liquid was directly added into Lysing Matrix E tubes for DNA extraction.

#### Stomach tubing

Rumen samples were collected from each calve in the morning between 8:00 and 10:00 am. at the end of the experimental trial on day 140 using an oral stomach tube modified according to Geishauser (1993) [46]. The instrument consisted of an oro-ruminal probe, a flexible tube and a manual suction pump. The probe was inserted orally into the ventral sac of the rumen and approximately 100 ml ruminal fluid sample was collected, while 200 ml of the fluid obtained at the beginning of sampling was discarded to avoid salvia contaminations. Rumen fluid samples were immediately frozen at − 80 °C until further analysis.

### DNA extraction

DNA from the rumen fluid and BS cell suspension was extracted using FastDNA™ SPIN Kit for Soil (MP Biomedical, Solon, OH, USA) with slight modifications in the manufacturer protocol as described previously [47]. The DNA extraction method included a bead-beating procedure to ensure effective cell lysis as proposed by [19]. The concentration and quality of DNA extracts was checked with NanoDrop 2000 spectrophotometer (Thermo Fisher Scientific, Waltham, MA, USA).

### PCR amplification and Illumina amplicon sequencing

The V1-V2 region of bacterial 16S rRNA gene was amplified with PCR and Illumina library was prepared as described previously [48]. A barcode (6-nt) and a linker (2-nt) sequence was added in the forward primer. Both primers were additionally linked to the overhang adapter sequences to make amplicons compatible with the Illumina MiSeq sequencing. The PCR mixture was the same as described by [9]. Thermocycling conditions for PCR involved a 3 min initial denaturation step at 95 °C, followed by 20 cycles including 10 s of denaturation at 98 °C, 10 s of annealing at 59 °C, 45 s of extension at 72 °C and 2 min final extension step at 72 °C. PCR product (1 *μ* l) was used in second PCR (15 cycles) step under same thermocycling conditions with reverse primer that contained additional sequence with integration of Illumina index primers and Illumina multiplexing sequence [48]. Amplicons were quality controlled by gel electrophoresis, purified and normalized with SequalPrep Normalization Kit (Invitrogen Inc., Carlsbad, CA, USA) and sequenced utilizing paired-end (2 × 250 bp) sequencing chemistry on an Illumina MiSeq platform. Sequences were submitted to European Nucleotide Archive under the accession number PRJEB41435.

### Bioinformatic analysis

The bioinformatic analysis of Illumina amplicon sequencing datasets covering V1-V2 region of 16S rRNA gene was done using QIIME 2 (2019.10) [49]. The paired-end (PE) Illumina raw sequences (2 × 250 bp) were imported in QIIME 2 using MultiplexedPairedEndBarcodeInSequence semantic type. The PE sequences were demultiplexed using cutadapt (v2.6) within QIIME 2 with q2-cutadapt plugin and demux-paired command, increasing the default error tolerance to 0.2. The residual artificial sequences such as barcodes, forward primer (22 bp) and reverse primer (19 bp) were trimmed by implementing cutadapt (v2.6) in QIIME 2 with q2-cutadapt plugin and trim-paired command [50]. The quality filtration step and joining of PE reads was done by implementing DADA2 pipeline in QIIME 2 with q2-dada2 plugin and denoise-paired command [51]. The trimmed PE sequences were quality filtered by retaining high quality bases (average quality score above 30) and PE reads were joined at a mean length of 313 ± 6 bp, chimeric sequences, non-overlapping regions and singletons were discarded and FeatureTable [Frequency] and FeatureData [Sequence] QIIME 2 artifacts were generated. The PE sequences from each sequencing run were processed separately throughout the analysis resulting in FeatureTable [Frequency] and FeatureData [Sequence] QIIME 2 artifacts per sequencing run after DADA2 step. The filtered FeatureTable [Frequency] artifacts were merged with qiime feature-table merge command and FeatureData [Sequence] artifacts with qiime feature-table merge-seqs command resulting in a total of 6,141,120 reads, with 23,262 ± 1758 reads (mean ± SEM) per sample. Taxonomic classification was performed with q2-feature-classifier plugin and classify-sklearn method using sklearn-based taxonomy classifier (pre-trained on SILVA reference database for 16S rRNA (release_132), under a default confidence of 0.7 [52, 53]. Sequences assigned to cyanobacteria and chloroplast as well as non-bacterial and unassigned sequences from FeatureData [Sequence] and FeatureTable [Frequency] artifacts were removed using q2-taxa plugin in QIIME 2 and a taxonomy-based filtering step using qiime taxa filter-seqs and qiime taxa filter-table commands. All low reads samples (< 5000 reads) were removed from FeatureTable [Frequency] and FeatureData [Sequence] artifacts with qiime feature-table filter-samples and qiime feature-table filter-seqs commands. A biom feature table (FeatureTable [Frequency]-with-taxonomy annotations) was produced with biom add-metadata command in QIIME 2 that was later converted into txt format with biom convert command. The feature table was filtered again by following strict criteria to remove the low abundance OTUs (≤ 0.2% of total reads per sample), thus, resulting in a total of 4,741,355 reads, with mean read counts for stomach tubing samples 17,716 ± 1590 and for buccal swab samples 21,014 ± 2014 (mean ± SEM) per sample and a total of 4906 unique bacterial OTUs. All unique bacterial OTUs were taxonomically reassigned using RDP database [54] and naïve Bayesian RDP classifier [55]. The output taxonomy table was filtered according to [56] with a defined confidence threshold cut-off value for each taxonomic level such as: genus (94.5%), family (86.5%), order (82.0%), class (78.5%) and phylum (75.0%) and the taxonomic assignments were omitted if they fall below the following sequence identity thresholds.

### Statistical analysis

Prior to any statistical analysis, the OTU count data was standardized with total sum normalization (TSS) method by dividing the OTU read counts by total reads in each sample. For alpha-diversity analysis, samples were rarefied to the lowest read counts in the dataset (a read depth of 1008; RS-dataset) and (a read depth of 1157; OS-dataset). Principal coordinates analysis (PCO/PCoA) was performed on standardized OTU abundance data, using Bray-Curtis dissimilarity matrix in Primer-e (PRIMER 6.1.16 and PERMANOVA+ 1.0.6 [57]; to visualize the samples clustering within specific group and between groups (sampling method, calf age and weaning). Bray-Curtis dissimilarity is a coefficient with value bound between 0 and 1, where 0 represents high similarity between two samples (share all species, with same abundance) and 1 represents no similarity between the two samples (share no species) [58]. Analysis of similarities (ANOSIM) test was performed in Calypso v8.84 [59] to confirm statistically significant differences between groups. ANOSIM statistic *R* corresponds to the mean rank differences between and within groups, with value between − 1 and + 1, where 0 represents complete random grouping [60]. To access how well the RS microbiota in BS samples represented that in rumen, scatter plots were generated using “pairs” function in R v4.0.3 [61]. A linear regression line to the scatter plots was added using R “abline” function. Pairwise taxonomy comparisons among sample types (rumen vs. buccal swabbing) were performed by calculating Spearman correlation coefficients using rcorr() function from “Hmisc” package in R. In order to further elucidate the extent to which the rumen composition similarities between the animals’ rumen samples in day 140 were reflected by their oral microbiome, a Bray-Curtis dissimilarity matrix was calculated for day 140 rumen samples and another Bray-Curtis dissimilarity matrix for day 140 RS portion of the BS samples and the two dissimilarity matrices were examined for correlation using Mantel test in R. The same animals were selected for both matrices (36 animals for day 140R and the same 36 animals for day 140BS) and both OTU abundance datasets were normalized separately to account for a total of 100% relative abundance in each sample before calculation of Bray-Curtis dissimilarity matrices. For OS-dataset, the standardized relative abundance table of bacterial genus-level taxa was scaled by row to generate heat maps using “gplots” package in R based on Spearman correlation and hierarchal average linkage clustering method. The microbial composition at phylum- and genus-level as well as alpha-diversity index was compared between groups using Kruskal–Wallis test and Dunn’s post hoc test in R. For Dunn’s statistical test, Benjamini–Hochberg algorithm [62] was used for *p*-value adjustment into false discovery rate (FDR). The FDR-adjusted *p*-values were considered significant at a probability of *p* < 0.05. The correlation matrix between bacterial alpha-diversity indices of earlyC and lateC groups and their body growth parameters (hip height, withers height, back length, body length, heart girth and live weight gain) was built based on the Spearman correlation coefficients.

## Supplementary Information

**Additional file 1: Figure S1**. Shannon index of RS bacterial communities in R and BS samples of different age group calves. **Figure S2**. Overlap of RS-OTUs covering V1-V2 region of bacterial 16S rRNA. **Figure S3**. Average relative abundances of RS bacterial phylum- and genus-level taxa in rumen and buccal swab samples of different age group calves. **Figure S4**. **a** Principal coordinates analysis plot of bacterial communities in 186 BS samples of different age group calves, after exclusion of potential RS taxa by mathematical filtering approach. **Figure S5**. Average relative abundances of OS bacterial genus-level taxa in BS samples of different age group calves. **Figure S6**. Shannon index of OS bacterial communities in BS samples of different weaning groups of calves. **Figure S7**. Bar-plot depicting within group similarity (mean and standard deviations) along the time. **Figure S8**. The Spearman correlation coefficients (R-values) between OTU’s relative abundance along the d140R samples with its abundance over the RS portion of the d140BS samples.

**Additional file 2 **: **Table S1**. Average relative abundance of RS bacterial taxa in samples of different age group calves. **Table S2**. Core RS bacterial OTUs in R and BS samples of different age group calves. **Table S3**. ANOSIM analysis between weaning groups. **Table S4**. Average relative abundances of RS bacterial taxa in BS samples of different weaning groups. **Table S5**. Average relative abundance of OS bacterial taxa in BS samples of different age group calves. **Table S6**. Average relative abundances of OS bacterial taxa in BS samples of different weaning groups. **Table S7**. Correlation matrix between bacterial alpha-diversity indices of earlyC and lateC groups and their body growth parameters. **Table S8.1**. Rare taxa OTUs in BS and R samples. **Table S8.1**. Mean, median and standard deviations of rare genus-level taxa in BS and R samples. **Table S9**. Mantel test statistics showing correlation between Bray-Curtis dissimilarity matrices of d140R samples and day 140 RS portion of the BS samples.

## Data Availability

Sequences were submitted to European Nucleotide Archive under the accession number PRJEB41435.
