# Supplementary material for: Evolution of rumen and oral microbiota in calves is influenced by age and time of weaning
Source: Anim Microbiome. 2021 Apr 21;3:31. doi: 10.1186/s42523-021-00095-3 (PMC8059317; doi:10.1186/s42523-021-00095-3)
Supplement: Supplementary file 1 — Additional file 1: Figure S1. Shannon index of RS bacterial communities in R and BS samples of different age group calves. Figure S2. Overlap of RS-OTUs covering V1-V2 region of bacterial 16S rRNA. Figure S3. Average relative abundances of RS bacterial phylum- and genus-level taxa in rumen and buccal swab samples of different age group calves. Figure S4. a Principal coordinates analysis plot of bacterial communities in 186 BS samples of different age group calves, after exclusion of potential RS taxa by mathematical filtering approach. Figure S5. Average relative abundances of OS bacterial genus-level taxa in BS samples of different age group calves. Figure S6. Shannon index of OS bacterial communities in BS samples of different weaning groups of calves. Figure S7. Bar-plot depicting within group similarity (mean and standard deviations) along the time. Figure S8. The Spearman correlation coefficients (R-values) between OTU’s relative abundance along the d140R samples with its abundance over the RS portion of the d140BS samples. [file 42523_2021_95_MOESM1_ESM.docx]

**Supplementary Information**

**Evolution of rumen and oral microbiota in calves is influenced by age and time of weaning**

Nida Amin^1^, Sarah Schwarzkopf^1^, Asako Kinoshita^1,2^, Johanna Tröscher-Mußotter^1^, Sven Dänicke^2^, Amélia Camarinha-Silva^1^, Korinna Huber^1^, Jana Frahm^2^, Jana Seifert^1^*

1 Institute of Animal Science, University of Hohenheim, Stuttgart, Germany

2 Institute of Animal Nutrition, Friedrich-Loeffler-Institut, Federal Research Institute for Animal Health, Bundesallee 37, 38116 Braunschweig, Germany

*corresponding author

Jana Seifert

University of Hohenheim

Institute of Animal Science

Emil-Wolff-Str. 6-10

70593 Stuttgart, Germany

[jseifert@uni-hohenheim.de](mailto:jseifert@uni-hohenheim.de)


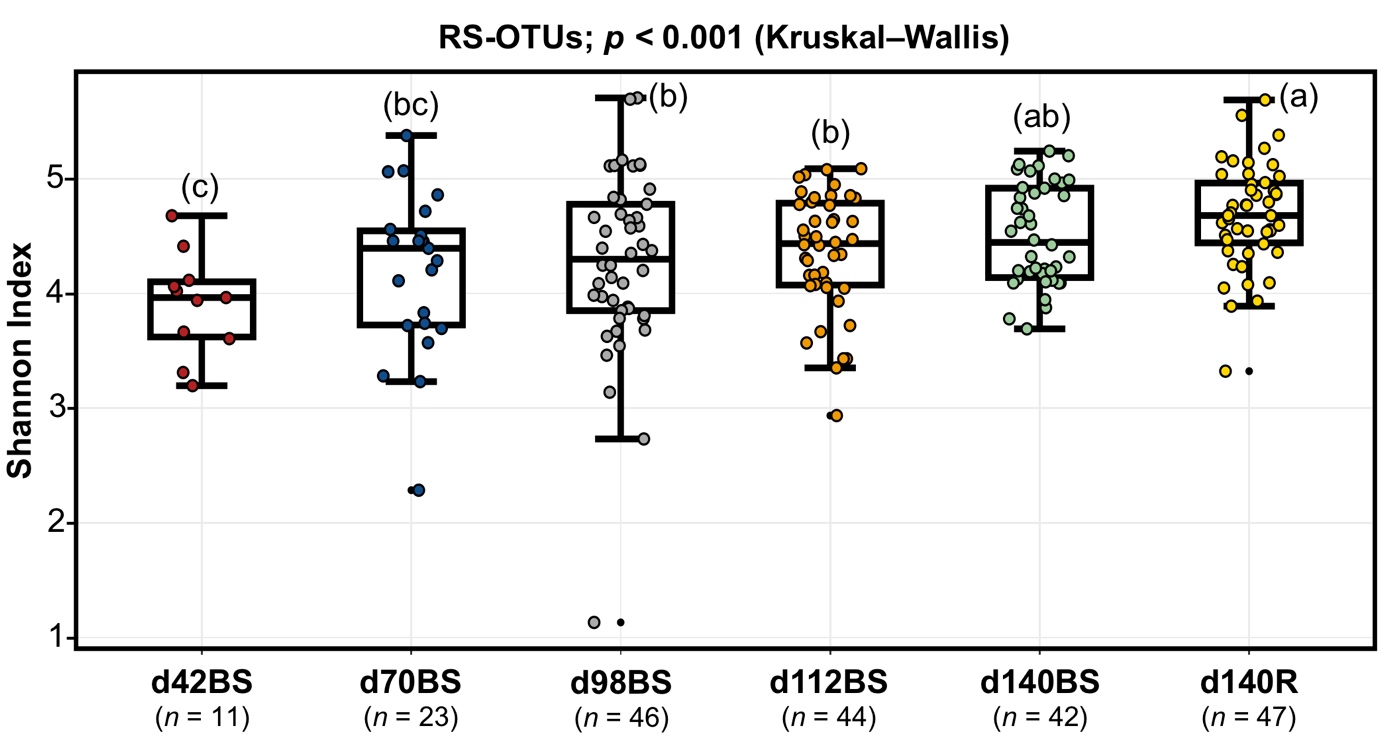


**Figure S1** Shannon index of RS bacterial communities in R and BS samples of different age group calves. Different age groups are indicated by different colours. Age groups that share superscript letters are not significantly different (*p* > 0.05, Dunn’s test).


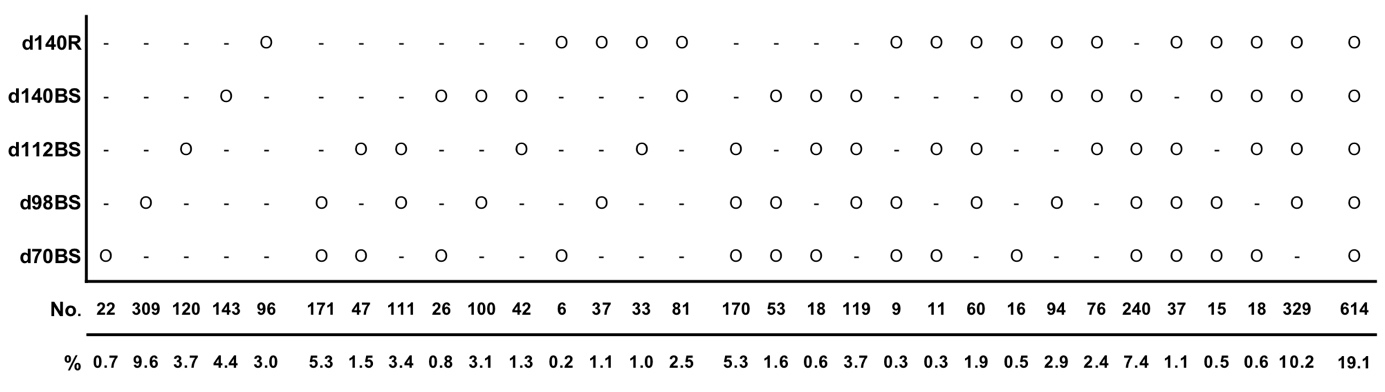


**Figure S2** Overlap of RS-OTUs covering V1-V2 region of bacterial 16S rRNA. A total of 3,425 RS-OTUs identified in the R and BS samples collected from 70, 98, 112 and 140-day-old calves were included in the dot plot. The number and percentage of shared OTUs are represented on x-axis and sampling day on y-axis.

**Figure S3** Average relative abundances of RS bacterial taxa at the phylum-level (a), and genus-level (b) in rumen (R) and buccal swab (BS) samples of different age group calves. Each bar represents an average value for animals at each age group: day 42 (11 animals), day 70 (23 animals), day 98 (46 animals), day 112 (44 animals), day 140 (42 animals) and day 140 rumen (47 animals). Taxa are shown as colored segments in bars.

**a**

**
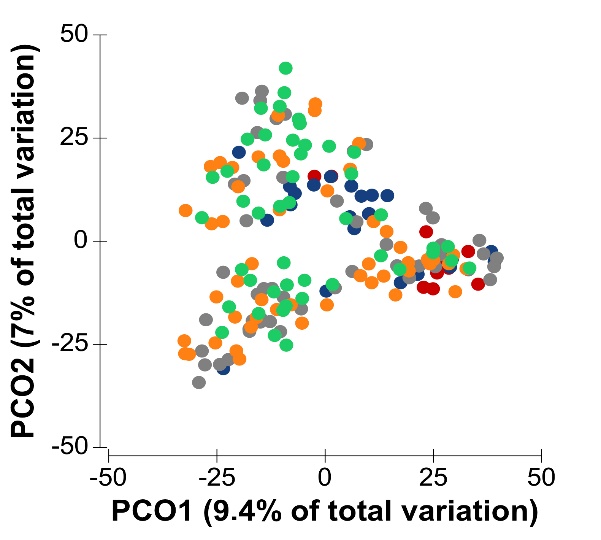
**

**Figure S4** **a** Principal coordinates analysis plot of OS bacterial communities in 186 BS samples of different age group calves, after exclusion of potential RS taxa by mathematical filtering approach. Each point represents one sample. Different age groups are indicated by different coloured circles: red circles, day 42BS; blue circles, day 70BS; grey circles, day 98BS; orange circles, day 112BS, and green circles, day 140BS. **b** Average relative abundances of OS bacterial taxa at the phylum-level.

**Figure S5** Average relative abundances of OS bacterial genus-level taxa in BS samples of different age group calves. High relative abundances are indicated by red colours and low relative abundances by blue colours. Clustering was done using Spearman correlation and average linkage method. Genus level taxa with < 0.5% contribution to the overall community are not included.


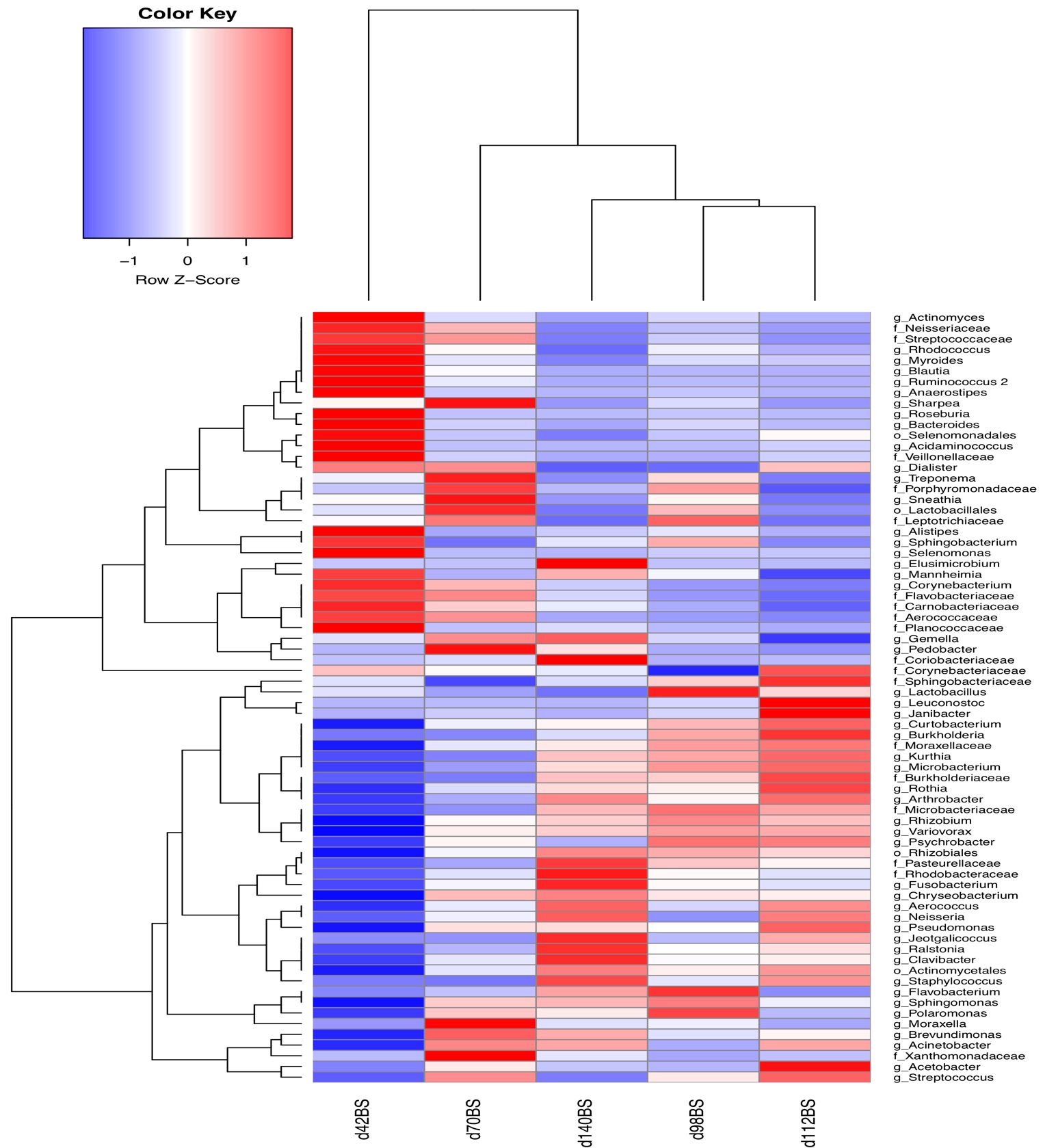

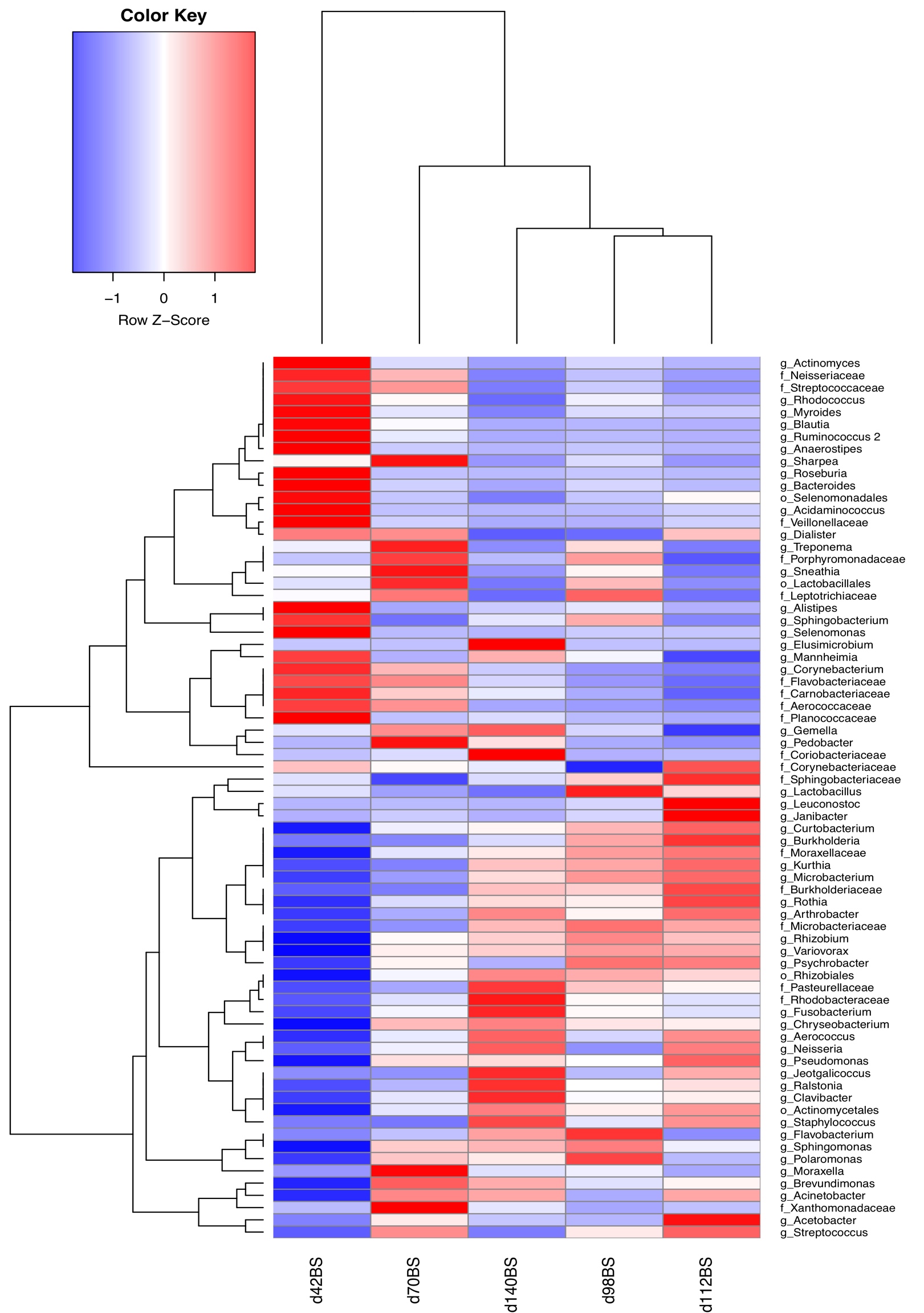


-1

0

1


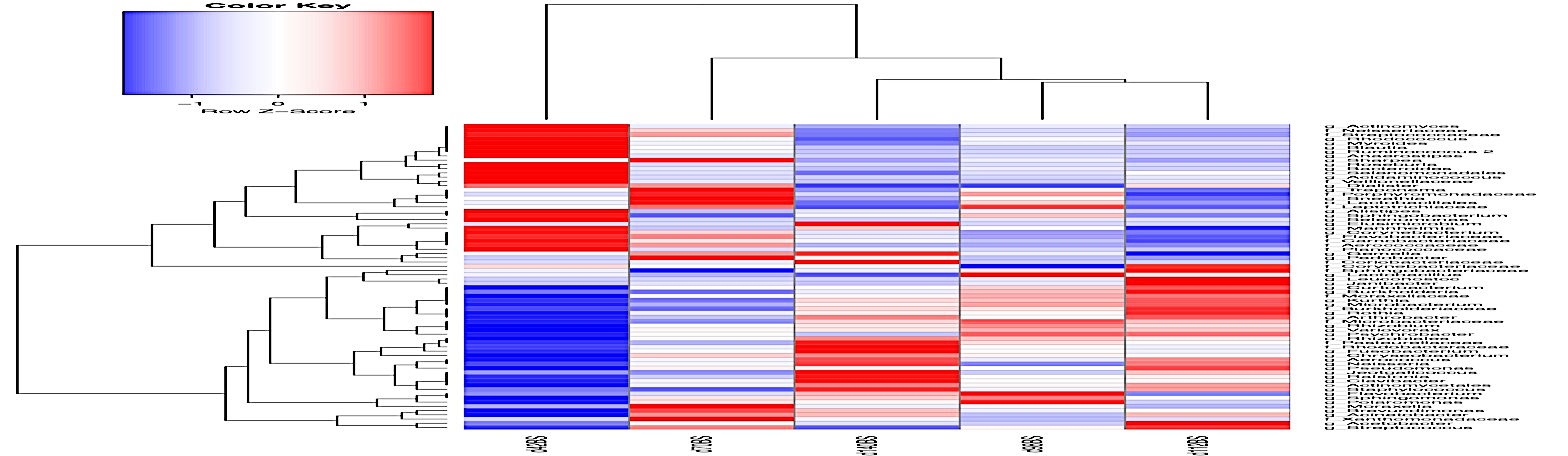


Row Z-Score

**d42BS**

**d70BS**

**d140BS**

**d112BS**

**d98BS**


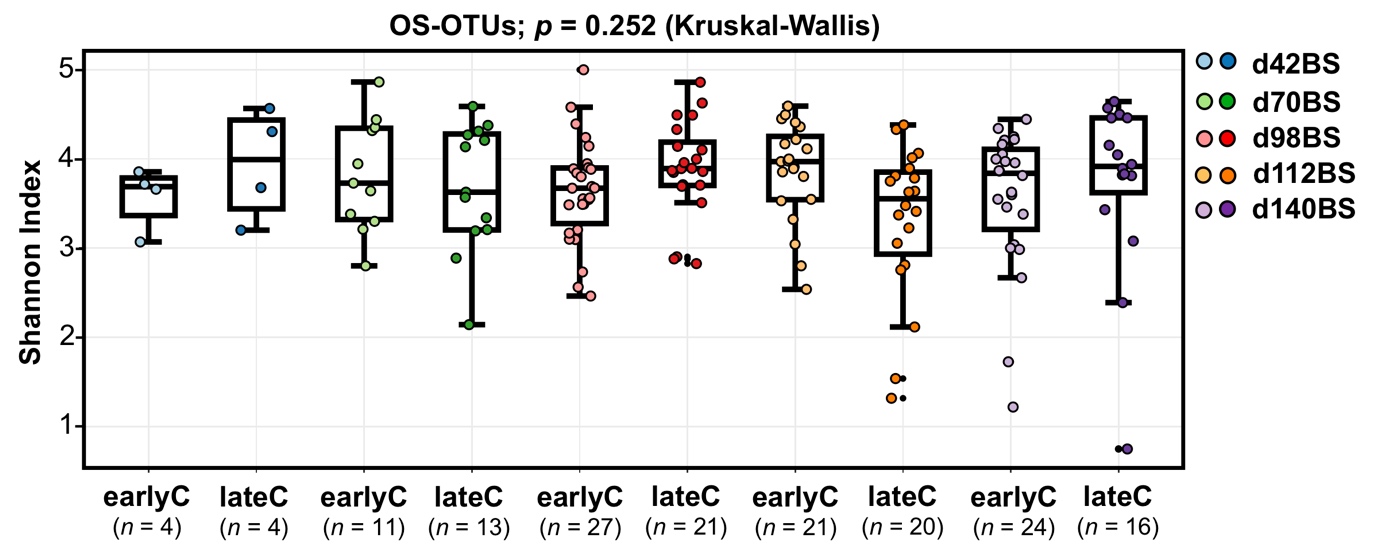


**Figure S6** Shannon index of OS bacterial communities in BS samples of different weaning groups of calves. Different weaning periods within each age group are shown by different colours.


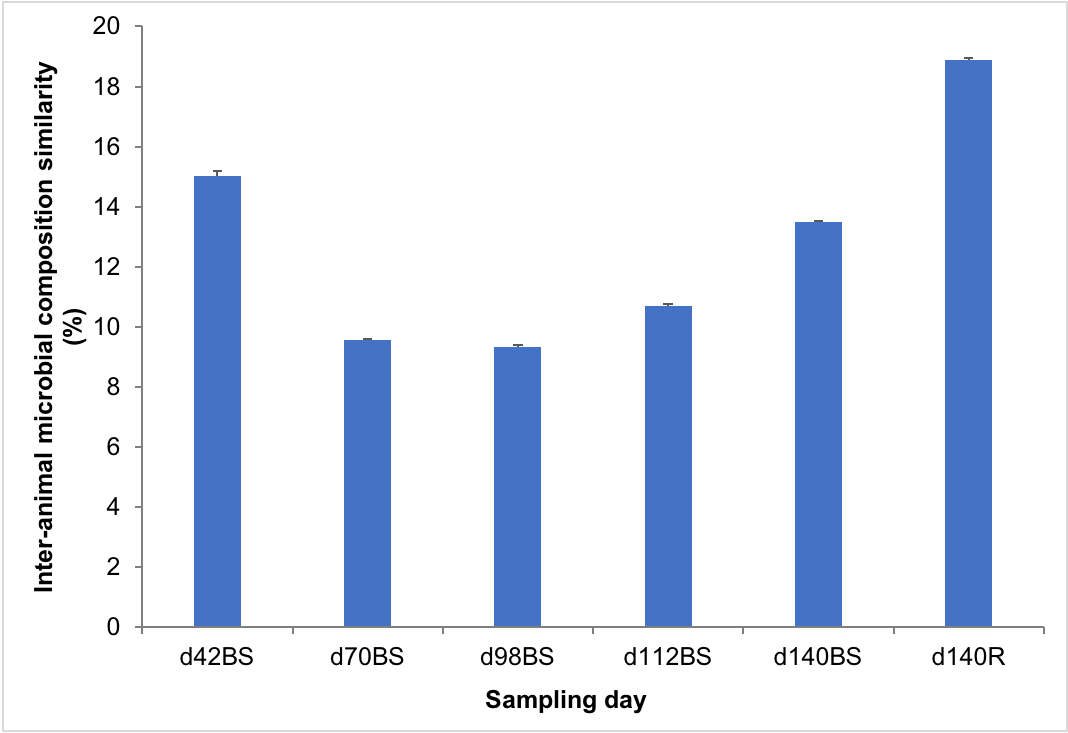


**Figure S7** Bar-plot depicting within group similarity (mean and standard deviations) along the time. Sampling day on the x-axis and percentage of inter-animal microbial composition similarity on the y-axis.

**Figure S8** Spearman correlation coefficients (R-values) between OTU's relative abundance along the d140R samples with its abundance over the RS portion of the d140BS samples.
